# Higher VO_2_max is associated with thicker cortex and lower grey matter blood flow in older adults

**DOI:** 10.1038/s41598-021-96138-5

**Published:** 2021-08-18

**Authors:** Gaia Olivo, Jonna Nilsson, Benjamín Garzón, Alexander Lebedev, Anders Wåhlin, Olga Tarassova, Maria M. Ekblom, Martin Lövdén

**Affiliations:** 1 Department of Psychology, University of Gothenburg, Haraldsgatan 1, 413 14 Göteborg, Sweden; 2 Aging Research Center (ARC), Department of Neurobiology, Care Sciences and Society (NVS), Karolinska Institutet, Stockholm, Sweden; 3 The Swedish School of Sport and Health Sciences, Stockholm, Sweden; 4 Department of Clinical Neuroscience, Karolinska Institutet, Stockholm, Sweden; 5 Department of Radiation Sciences, Umeå University, Umeå, Sweden; 6 Umeå Center for Functional Brain Imaging (UFBI), Umeå University, Umeå, Sweden; 7 Department of Neuroscience, Karolinska Institutet, Stockhom, Sweden

**Keywords:** Neuroscience, Cognitive ageing, Neuro-vascular interactions

## Abstract

VO_2_max (maximal oxygen consumption), a validated measure of aerobic fitness, has been associated with better cerebral artery compliance and measures of brain morphology, such as higher cortical thickness (CT) in frontal, temporal and cingular cortices, and larger grey matter volume (GMV) of the middle temporal gyrus, hippocampus, orbitofrontal cortex and cingulate cortex. Single sessions of physical exercise can promptly enhance cognitive performance and brain activity during executive tasks. However, the immediate effects of exercise on macro-scale properties of the brain’s grey matter remain unclear. We investigated the impact of one session of moderate-intensity physical exercise, compared with rest, on grey matter volume, cortical thickness, working memory performance, and task-related brain activity in older adults. Cross-sectional associations between brain measures and VO_2_max were also tested. Exercise did not induce statistically significant changes in brain activity, grey matter volume, or cortical thickness. Cardiovascular fitness, measured by VO_2_max, was associated with lower grey matter blood flow in the left hippocampus and thicker cortex in the left superior temporal gyrus. Cortical thickness was reduced at post-test independent of exercise/rest. Our findings support that (1) fitter individuals may need lower grey matter blood flow to meet metabolic oxygen demand, and (2) have thicker cortex.

## Introduction

Cardiorespiratory fitness has been associated with individual differences in cognitive performance and brain integrity in older adults^1,2^. Regular (i.e. chronic) physical exercise has been associated with greater total grey matter volume (GMV)^3,4^, greater volume of the hippocampus^3,5–8^, and larger volume in more than 80% of all cortical regions^5,7,9^. Most of the above investigations have used valid measures of cognitive performance and brain volumes, but settled for feasible though rather imprecise measures of cardiorespiratory fitness. More direct measures of cardiorespiratory fitness can be achieved with measurement of the maximum amount of oxygen (VO_2_max) that an individual can utilize during maximal exercise^10^. VO_2_max has also been related with cortical thickness (CT) in frontal, temporal and cingular cortices^11^, as well as in the insula and precuneus^12^. The positive association between cardiovascular fitness and brain morphology was deemed to be more specific to the older rather than younger age^13^. However, a recent study in a large middle-aged adult sample (n = 2,103) reported a positive association of VO_2_max with total brain volume and GMV of the middle temporal gyrus, hippocampus, orbitofrontal cortex and cingulate cortex in the general population^14^. VO_2_max-associated greater GMV in the dorsal striatum has been proposed to be responsible for the better cognitive flexibility observed with higher VO_2_max.

Several different mechanisms have been so far proposed to explain the virtuous relationship between cardiovascular fitness and brain health in aging. Post-exercise astrocyte swelling, blood flow increases, and increases in the level of Brain-Derived Neurotrophic Factor (BDNF), a neurotrophin strongly implicated in brain plasticity and capable of inducing rapid changes in CT and synaptic density, have all been suggested to mediate the beneficial effects of fitness on neuroplasticity^15–17^. Indeed, a single session of 35 min of physical activity can already raise the levels of BDNF^18,19^. Transient positive effects on cognitive performance have also been reported already after single bouts of physical activity^20–22^. Working memory and executive functions have been reported to be particularly improved by physical activity, with effects lasting up to two hours after the cessation of the exercise bout^23–25^. Whether rapid morphological changes in GMV and CT can be induced after single sessions of physical exercise is, however, an open issue, as most of the studies of brain structure have had intervention designs of at least 4 weeks of physical exercise^26^. Nonetheless, task-related changes in measures of grey matter (GM) are known to occur very rapidly and can be detected already over hours or even a few minutes^27–29^. These effects might be mediated by rapid changes in blood flow^30^. Indeed, arterial blood has larger T1 (relaxation time) than brain tissues, therefore substantial perfusion changes can induce apparent tissue changes in the T1-weighted images^30^ that are typically used to assess brain volume and cortical thickness.

Fluctuations in cerebral blood flow have also been proposed to explain the changes in brain activity after single sessions of physical exercise^16,17^. Previous research suggested that improvements in performance following a single session of physical exercise are coupled with increases in the magnitude of functional brain activity during executive tasks mainly in frontal areas^20,24,31^; however, there are also reports showing that task-related functional activity in several other areas, such as the cerebellum^25,32^, hippocampus^31,32^, parietal cortex^32^, and insula^20^ is increased following acute physical exercise. Moreover, the exercise-induced changes in functional brain activity and executive performance remain unknown. These lacunae, as well as the variability in study protocols and the small number of studies available, warrant further studies on the short-term effect of physical exercise of brain activity^33^.

Aerobic fitness is likely to exert a regulatory role on cerebral blood flow in aging^34^, by modulating myocardial function^35^, leading to lower arterial stiffness^36^ and ultimately improving cerebral blood flow^36,37^. Accordingly, VO_2_max has been positively related with higher middle cerebral arteries compliance^38,39^ and with higher regional cerebral blood flow in patients with coronary artery disease^40^. By sustaining aerobic capacity, aerobic fitness may thus counteract the detrimental effect of aortic stiffness on cognitive aging^41^. However, the findings have been so far conflicting. One study reported the existence of a positive relationship between fitness and cerebral blood flow in females but not in males^42^. Another study did not detect any association of cerebrovascular reactivity or blood flow with VO_2_max^43^, and another reported even a negative relationship with VO_2_max^38^. A potential explanation for these discrepancies is that the positive effect of aerobic fitness on age-related decline may go beyond vascular effects and more directly involve neurons’ preservation and metabolism^2^, as suggested by the positive correlation between NAA levels (N-acetyl-aspartate, a brain-specific metabolite enhancing mitochondrial energy production) and cardiovascular fitness and working memory^2^.

The present study investigates the effect of acute physical exercise on the brain and cognition in older adults, and follows our previous work focusing on cognitive performance and cerebral blood flow^44^. In our former study, we found that grey matter blood flow (GMBF) (assessed with arterial spin labelling) decreased after 30 min of cycling at moderate intensity compared with 30 min of rest. Working memory performance, on the other hand, was unaffected by the intervention. In the present ancillary work, we focus on the effects of acute physical exercise, compared with rest, on grey matter measures (GMV, CT) and task-related brain activity in older adults. We hypothesized that the exercise group would exhibit larger GMV and mean CT, as well as higher task-related activity post-intervention, compared with the relaxation group. We also hypothesized that changes in brain activity and GM measures would partly depend on the underlying changes in GMBF. Additionally, we hypothesized that VO_2_max would be positively related with working memory performance, mean GMBF and GM morphological measures. The study was preregistered on the Open Science Foundation platform (https://osf.io/bxjkq; https://osf.io/jg8tz; https://osf.io/4mxgt).

## Methods

### Subjects

The study was approved by the ethics committee of region Stockholm in Sweden (Regionala Etikprövningsnämnden i Stockholm, Dnr: 2018/1340–31/2) and complied with the principles of the Declaration of Helsinki. Participants were recruited via advertisements in local newspapers and flyers in the Stockholm area between September and December 2018. The ads sought older volunteers, aged between 65 and 75 years, for a study investigating the effects of physical exercise and rest on brain function Inclusion criteria were: availability to attend all sessions; adequate hearing; normal or corrected vision; fluent Swedish (to ensure the understanding of the instructions); adequate mobility to ensure a safe performance of the physical exercise; right-handedness (to avoid handedness-related variability in brain structure and motor-related activity during the task^45–49^); weight below 120 kg. Exclusion criteria were: previous or current neurological diseases; a score below 26 on the Mini-Mental State Examination (MMSE)^50^; previous or current cardiovascular diseases (blood pressure up to 200/100 mmHg was allowed); history of brain damage or stroke; uncontrolled metabolic diseases; type I or pharmacologically-treated type II diabetes; current cancer, unless more than one year had passed since the treatment end; psychiatric illness, except for a history of mild to moderate depression and/or anxiety; history of head trauma resulting in loss of consciousness; previous participation in studies involving cognitive tests; presence of metal in the body; claustrophobia; sound sensitivity; neuromotor or musculoskeletal dysfunctions; cardiovascular-active medications; ongoing infections; chest pain. An introduction meeting was held at the Aging Research Center (Karolinska Institute, Stockholm) for eligible participants. Of the 73 individuals who had initially expressed their interest in the study, 50 joined the experimental session. One was excluded due to MRI signs suggestive of past, undiagnosed stroke. Recruitment and screening procedures have been described in more detail elsewhere^44^ (https://osf.io/bxjkq). The exercise group included 24 individuals, while the relaxation group consisted of 25 individuals. Demographic variables, physical activity, MMSE score, blood pressure, and subjective memory evaluation of the two groups were comparable (table S6, Appendix D, Supplementary Material).

### Behavioural and demographic measures

In the introduction meeting, participants received detailed information concerning the study and signed the informed consent. Participants were blind to the hypothesis of the study (i.e. whether we expected moderate intensity physical exercise to be superior to rest in improving cognition and brain imaging measurements) in order to minimize the influence of participants’ expectations on the cognitive performance measurements. Demographic information was collected. All participants were administered the Edinburgh Handedness Inventory to confirm right-handedness and the Mini Mental State Examination (MMSE) to assess their cognitive status. The International Physical Activity Questionnaire (IPAQ)^51^ was used to estimate their habitual physical activity. Blood pressure was measured. Participants were also provided with instructions on how to perform the n-back task to be done in the main experimental session, and could try it out for 5 min.

### Fitness assessment and calculation of work rate to be used during exercise

Fitness characterization was performed at the Åstrand Laboratory at the Swedish School of Sport and Health Sciences, GIH. Participants performed a submaximal incremental test on a cycle ergometer and a maximal incremental running test on a treadmill^44^. The submaximal test on the bike was used to identify the individual work rate and heart rate during biking at which the VO2 reached around 60% of the VO2max recorded during the maximal running test. This work rate is expected to result in a perceived level of exertion from 13–15 on the Borg RPE scale and a heart rate of around 60–70% of maximal individual heart rate. This individual work rate and heart rate was then used during biking in the exercise intervention of the experimental session.

The submaximal test was performed on a cycle ergometer (model 828E, Monark, Varberg, Sweden). The protocol started with 4 min cycling at a standard work rate (resistance of 0.5 kp, 60 revolutions per minute), after which the resistance was increased in steps of 0.5–1 kp, until reaching 60–80% of individual maximal capacity and a rate of perceived exertion (RPE) of around 16 (and not above 16). The maximal incremental running test on a treadmill was used to measure VO2 max, the gold standard for the metabolic measurement in applied human research. Participants warmed up for 5–10 min before starting the test. The treadmill was initially set to an incline of 1 degree and a comfortable speed (around RPE 12–13). Incline degree and/or speed were increased every minute until volitional exhaustion. VO2 max was measured using a computerized metabolic system (Jaeger Oxycon Pro, Hoechberg, Germany). The highest 30 s of registered values of VO2 were referred to as VO2 max. All procedures have been described in more detail elsewhere^44^ (https://osf.io/bxjkq).

### Experimental session and MRI acquisition

The experimental session was performed at the Siemens Prisma 3-Tesla facility at the Karolinska University Hospital in Huddinge. Subjects randomized to the exercise group cycled on a stationary bike for 30 min, starting at the intensity estimated from the fitness characterization. Subjects randomized to the relaxation group were asked to lay down and relax for 30 min, while listening to relaxing music with water-sounds in the background. MRI scanning was carried out at pre-test, and 7 min after the exercise or rest session. This time was necessary to allow for the safe placement of the participants back in the scanner. GMBF was measured with an arterial spin labeling (ASL) acquisition (TR 4000 ms; TE = 12 ms; inversion time (TI) = 2000 ms; FOV 128 × 128; 1.9 × 1.9x4.5 mm^3^; slice thickness 4.5 mm; 32 slices). FMRI was acquired with a T2*-weighted echoplanar imaging sequence (TR = 2000 ms; TE = 30 ms; flip angle = 90°; slice thickness = 3.5 mm; voxel-size 3.5 mm^3^; slice spacing = 3.5 mm; 38 slices). Structural images were acquired via a MPRAGE (T1-weighted magnetization prepared gradient-echo) sequence (Repetition time (TR) = 2300 ms; Echo time (TE) = 2.01 ms; flip angle = 9°; field of view (FOV) 240 × 256; voxel sixe: 1 mm^3^; slice thickness 1 mm; 208 slices).

### fMRI protocol

**Figure 1:**
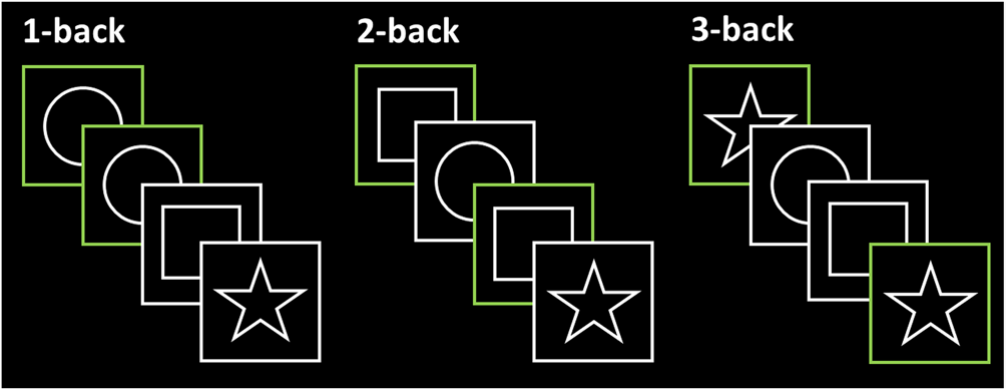
Figural n-back task. The task consisted in alternating blocks with different cognitive load (1-back, 2-back, 3-back), administered three times each. Each block lasted one minute.

A figural n-back task was used to assess working memory performance during the fMRI (Fig. 1). The n-back task is a widely used task to measure working memory^52^, which is part of the executive functions domain. Executive functions are particularly sensitive to the effects of physical exercise^20,24,31,53,54^. The n-back task is one of the most used paradigms to assess executive (specifically, working memory) performance, and it is largely employed in brain imaging research given its easy implementation in the scanner environment^52^. The task consisted in alternating blocks with different cognitive load (1-back, 2-back, 3-back), administered three times each. In each block, the subjects were presented with a sequence of stimuli, and they were instructed to indicate when the current stimulus matched the one from *n* steps earlier in the sequence. Between blocks a 4-s fixation screen was displayed, indicating the rule for the forthcoming level. Each block lasted one minute. The accuracy on the task was calculated as: (correct hits + correct rejections)/total stimuli. The accuracy on each load and average accuracy across loads were calculated. Prior to pre-test imaging, a short practice (10 min) of the n-back task was performed when the subject was in the scanner. Due to a malfunctioning of the keyboard, n-back data of nine participants could not be recorded. Two extreme outliers on n-back performance were identified and excluded from the analyses^44^.

### Pre-processing of functional imaging data

The pre-processing of fMRI data is described in Appendix A, Supplementary Materials. Briefly, fMRI pre-processing steps for the BOLD signal analysis were carried out with Statistical Parametric Mapping 12 (SPM 12) and DPARSFA (http://rfmri.org/DPARSF) and included slice-timing, realignment, coregistration with the structural images, normalization, resampling to a 3 mm^3^ voxel-size and smoothing with an 8 mm full width at half maximum (FWHM) Gaussian kernel. As fMRI signal can be confounded by individual differences in the neurovascular coupling, especially in aging^55^, we additionally performed a network analysis with a graph theory approach, less sensitive to such confounds^55^. These analyses were included in the preregistration (https://osf.io/jg8tz), and are described in Appendix B, Supplementary Materials. Results are reported in figures S1, S2 and tables S1, S2 in Appendix B, Supplementary Materials.

### Pre-processing of structural imaging data

Structural data were pre-processed with CAT12 (http://www.neuro.uni-jena.de/cat/). The cross-sectional pipeline was initially used to process data for the cross-sectional, correlational part of this project. The longitudinal pipeline was used for the longitudinal assessment. This consisted in: an initial inverse-consistent rigid registration; segmentation into GM, white matter and cerebrospinal fluid probability maps; non-linear spatial registration and averaging of the images; modulation; and smoothing with a 8 mm FWHM Gaussian kernel. Two data-points (post-test) presented with severe ring artefacts at visual inspection and were thus excluded from further analysis. Total GMV was calculated from the non-modulated image of the remaining subjects. CT and surface reconstruction were obtained from CAT12 as part of the longitudinal pipeline with a projection-based thickness (PBT) approach. Briefly, tissue segmentation is used to estimate the white matter distance, then local maxima (corresponding to the cortical thickness) are projected onto other GM voxels using a neighbouring algorithm depending on the white matter distance. Smoothing with a 12 mm FWHM Gaussian kernel was then applied to the surface images. More details on the pre-processing of structural imaging data can be found in Appendix A, Supplementary Material.

### Pre-processing of ASL data

ASL data were checked for movement by calculating the root mean square signal change (DVARS) values, defined as the root mean square intensity difference of paired volumes (volume N to volume N + 1)^46^. Data-point with DVARS values exceeding the threshold of 75th percentile + 1.5 times the InterQuartile Range (IQR) were excluded due to excessive movement. One subject was excluded from the analysis as more than 30% of the acquisition was flagged for excessive movement (pre-test). ASL data were pre-processed with FSL (FMRIB Software Library). The oxford_asl command included in the BASIL (Bayesian Inference for Arterial Spin Labeling MRI) toolbox was used to process ASL data^56^. This automated pipeline includes motion correction, followed by the creation of subject-space tissue perfusion maps that are subsequently registered to the MNI standard space by applying the parameters generated during the registration of the subject’s structural image (see Appendix A, Supplementary Material). The calibrated perfusion map is then generated using the proton density weighted image, using the cerebrospinal fluid (CSF) as reference to calculate the equilibrium magnetisation of blood within a CSF mask. Calibrated perfusion images express GMBF in ml/100 g/min. The maps were smoothed with an 8 mm FWHM gaussian kernel. Prior to statistical analyses, all images were visually inspected to ensure the good quality of the pre-processing.

## Results

Preregistered hypotheses (H_i_) and follow-up research questions (RQ_i_) are described below, along with the analyses used to address them (https://osf.io/bxjkq; https://osf.io/jg8tz; https://osf.io/4mxgt). As per pre-registration, the generalized-linear mixed model analyses for the longitudinal part of the project were performed with Statistical Package for Social Science (SPSS); cross-sectional correlational analyses were performed with R Studio. All hypotheses, follow-up research questions and corresponding analyses were pre-registered prior to the study (https://osf.io/bxjkq; https://osf.io/jg8tz; https://osf.io/4mxgt), regardless of their inclusion in the main text or in the Supplementary Materials. However, given the nature of the findings, some hypotheses became less meaningful, and were not expected to lead to the a priori hypothesized results. These hypotheses and corresponding analyses are reported in Appendix C, Supplementary Materials. Results are reported in tables S3, S4, S5 in Appendix C, Supplementary Materials.

### Longitudinal analyses: effect of a single session of exercise vs rest on imaging measures

#### Effects of exercise on grey matter volume

##### **H1**

Total grey matter volume will increase after exercise compared with rest.

We used a generalized-linear mixed model with gamma distribution and robust estimation to test for effects of the group, session, and the group*session interaction (that directly tests our hypothesis) on total GMV. A random intercept model was built, where subjects represented the random factor, group was treated as between-subject factor, and session was treated as within-subject factor. The residual method was used for the estimation of denominator degrees of freedom. The threshold for significance was set at p < 0.05.

Group, session and the group*session interaction had no statistically significant effect on total GMV (table S7 and figure S3, Appendix D, Supplementary Materials).

##### **RQ1**

Is the effect of exercise on grey matter volume region-specific?

A flexible factorial model as implemented in SPM12 was used to test for main effects of the group, session, and group*session interaction on whole-brain, voxel-wise GMV. The flexible factorial model allows for mixed-model specification, where group was set as between-subject factor and session was set as within-subject factor. A preliminary uncorrected threshold of p < 0.001 was applied^57^. Voxels surviving such threshold were further corrected for family-wise error (FWE) rate at cluster level with a threshold of p < 0.05^57^.

No significant effects of the group, session, or group*session on voxel-wise GMV were found, indicating no effect of physical activity on GMV.

#### Effects of exercise on cortical thickness

##### **H2**

Mean cortical thickness will increase after exercise compared with rest.

Mean CT was not normally distributed according to the Shapiro-Wilks’s test for normality, nor was its log transformation. Thus, we applied a rank transformation to normal scores according to Rankit’s formula for proportional estimation: (r-1/2)/w ; where w is the number of observations and r is the rank, ranging from 1 to w. Rankit’s formula has been deemed to be more accurate than Blom, Tukey or Van der Warden^58^. A generalized-linear mixed model with robust estimation was used to test for effects of group, session, and group*session interaction (critical effect to test our hypothesis) on mean CT. Subjects represented the random factor (intercept model), group was entered as between-subject factor, and session was treated as within-subject factor. The residual method was used for the estimation of denominator degrees of freedom. The threshold for significance was set at p < 0.05.

No statistically significant effects of the group or session*group on mean CT were detected. Only the effect of session was statistically significant, indicating reduced mean CT at post-test independent of group (Cohen’s d = 0.114) (Table 1).

**Table 1:** Statistical results for the group, session, and group by session effects on mean CT (n = 49).

	C.E	S.E	t	Adj. Sig	C.I. 95% (min, max)
**Group, F(1,90) = .001; p = .991**					
Exercise vs Rest	.003	.276	.012	.991	− .546, .552
**Session, F(1,90) = 5.935; p = .017 **^**†**^					
Post-test vs Pre-test ^**†**^	− .111	.045	− 2.436	.017	− .201, − .020
Group*session F(1,90) = .379; p = .539					
Exercise, post- vs pre-test	− .083	.049	− 1.687	.095	− .180, .015
Rest, post- vs pre-test	− .139	.077	− 1.813	.073	− .291, .013

C.E., contrast estimate; S.E., standard error; df, degrees of freedom; C.I., confidence intervals.

^†^Statistically significant.

##### **RQ2**

Is the effect of exercise on cortical thickness region-specific, and is it associated to changes in GMBF?

CAT12 was used to test for a group*session [factors] effect on whole-brain, vertex-wise CT [dependent variable]. Group was treated as between-subject factor; session was entered as within-subject factor. A preliminary uncorrected threshold of p < 0.001 was applied^57^. Vertices surviving such threshold were further corrected for FWE rate at cluster level with a threshold of p < 0.05^57^.

**Figure 2:**
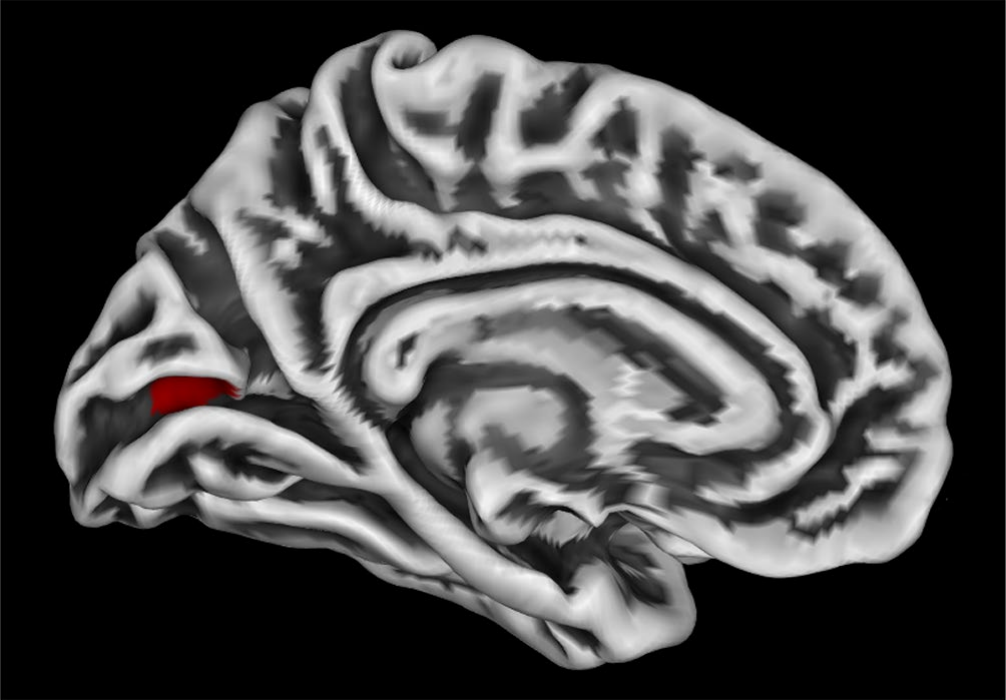
Cortical thickness increase in the visual cortex. The vertex-wise analysis revealed a cluster with pre- to post-test increased cortical thickness in the visual cortex (red) (p FWE-corr = .004). The figure was obtained with CAT12, overlaying the cluster on a surface rendering. MNI coordinates (x, y, z): −13, −77, 11. The Harvard–Oxford Cortical Structural Atlas was used to derive the location of the coordinates.

Despite the overall reduction in mean CT, this vertex-wise exploratory analysis revealed a statistically significant increase in CT in the intracalcarine (visual) cortex (p FWE-corr = 0.004; 136 voxels; t = 5.05; Fig. 2). No effects of the intervention or intervention*session were found.

The same analysis was carried out by correcting for the regional GMBF of the visual cortex. The results were unchanged.

##### **H3**

Pre- to post-test changes in total grey matter volume and mean cortical thickness will correlate with changes in GMBF more strongly in the exercise group.

The difference in mean cortical thickness between sessions was computed (post-test vs pre-test). A positive difference reflected a post-test increase in mean cortical thickness. One subject was an extreme outlier on the mean cortical thickness and was excluded from the correlation analysis. Effects of the group, GMBF, and GMBF*group effects were tested with a generalized linear model with robust estimation. The group*GMBF changes was the critical effect that addresses our analysis. The threshold for significance was set at p < 0.05.

Changes in GMBF was not significantly associated with changes CT and group did affect the magnitude of the association in a statistically significant way.

#### Effects of exercise on brain activity

##### **H4**

Exercise will increase task-related brain activity compared with rest.

SPM 12 was used to analyse fMRI data. First-level analyses were carried out to generate the 2-back vs 1-back and 3-back versus 1-back contrasts, correcting for motion parameters. A preliminary one-sample t-test was carried out to ensure that the task was eliciting the expected effect on the BOLD signal. A full factorial model was then run to test for the effects of group, session, load, and all the two- and three-ways interactions on voxel-wise whole-brain activity during the figurative working memory task. The group*session and group*session*load interactions were the effects of interest. Group was set as between-subject factor; session and load were treated as within-subject factors. Two load levels were entered: the 2- vs 1- back contrast was set as first level; the 3- vs 1-back contrast was set as second level. The threshold for significance was set at p < 0.001, further corrected for multiple testing with a family-wise error (FWE) correction at cluster level with a threshold of p < 0.05^57^. Post-hoc tests were performed masked for the relevant significant effects.

The one-sample t-test showed widespread fronto-parietal task-related activity relative to both the 2-back versus 1-back and 3-back versus 1-back contrasts (Figure S4, S5 and table S8, Appendix D, Supplementary Materials). No statistically significant effects of the group, session, group*session or group*session*load interaction were found. Thus, brain activity did not show statistically significant changes from pre- to post-test, and was unaffected by the type of the intervention.

### Cross-sectional analyses: associations between VO_2_max and pre-test cognitive and imaging measures

#### Association between VO_2_max and working memory

##### **H5**

VO_2_max will positively correlate with working memory performance.

ANOVA-repeated measures was used to test whether VO_2_max (independent variable) was associated with working memory performance (dependent variable). Prior to the analysis, n-back accuracy was tested for the presence of extreme outliers. One subject was an extreme outlier on the 1- and 2-back; another subject was an outlier on the 1-back task. No outliers were detected on the 3-back performance. Homogeneity of variance was inspected visually and formally tested with the Non-constant Variance (NCV) Score test (car package). Homogeneity of variance was confirmed on all loads (1-back, p = 0.744; 2-back, p = 0.253; 3-back, p = 0.969). Normality of the residuals was not met; however, given that ANOVA is quite robust to violations of the normality assumption^59,60^, repeated-measures ANOVA was applied. Nonetheless, we also analysed each load separately with generalized linear models with binomial distribution, selecting the number of correct answers as numerator and entering the total number of trials as denominator. All analyses were corrected by age and gender. A full factorial model (load, VO_2_max, load* VO_2_max) was carried out, where VO_2_max and load*VO_2_max were effects of interest. The threshold for significance was set at p < 0.05.

No statistically significant effect of VO_2_max or load*VO_2_max interaction on n-back accuracy was detected at the repeated-measure ANOVA, either at the uncorrected model nor when correcting for age and gender (table S5, Appendix C, Supplementary Material). When examining each load separately with generalized linear models, no significant associations between accuracy and VO_2_max were found at any load (p > 0.910).

#### Association between VO_2_max and grey matter blood flow

##### **H6**

VO_2_max will positively correlate with mean grey matter blood flow.

Prior to testing for an association between VO_2_max (independent variable) and mean GMBF (dependent variable), mean GMBF was tested for outliers. No extreme outliers were detected. Linearity of the relation and homogeneity of variance were visually verified. Additionally, the NCV test was applied to formally confirm homogeneity of variance (p = 0.266). The residuals approximated a normal distribution; however, gamma distribution was a better fit for mean GMBF (AIC = 308.082 vs AIC = 313.172). Therefore, a generalized linear model with gamma distribution (log-link) was used to test for associations between VO_2_max and mean GMBF. Age and gender were covariates in the analysis. The threshold for significance was set at p < 0.05.

VO_2_max displayed a statistically significant negative association with mean GMBF at the uncorrected model (p = 0.016; Confidence Intervals (CI) 95%: −0.04, −0.004) with a medium effect size (r = −0.35, corresponding to Cohen’s f = 0.37); a trend was still present after correcting for age and gender, though it only approached statistical significance (p = 0.08; r = −0.028, Cohen’s f = 0.29) (Table 2). This finding was in contrast with our prediction that VO_2_max would be positively associated with mean GMBF.

**Table 2:** Associations between VO_2_max and morphological measures (n = 49).

	Estimate	Std. Error	T	P	C.I. 95% (min, max)
GMBF	−.018	.010	−1.793	.08	− .038; .002
GMV	−.0006	.0005	1.192	.240	− .0016; .0004
CT	.005	.002	2.079	.043 *	**.001, .009**

Corrected for age and gender.

##### **RQ3**

Is VO_2_max related with regional-specific GMBF?

Linear regression was used to test for a correlation between VO_2_max (independent variable) and whole-brain, voxel-wise GMBF (dependent variable). The analysis was corrected for age and gender. The threshold for significance was set at p < 0.001; a further FWE-correction at p < 0.05 was applied to voxels surviving the preliminary uncorrected threshold^57^. Post-hoc analyses were masked for the main effect of VO_2_max.

**Figure 3:**
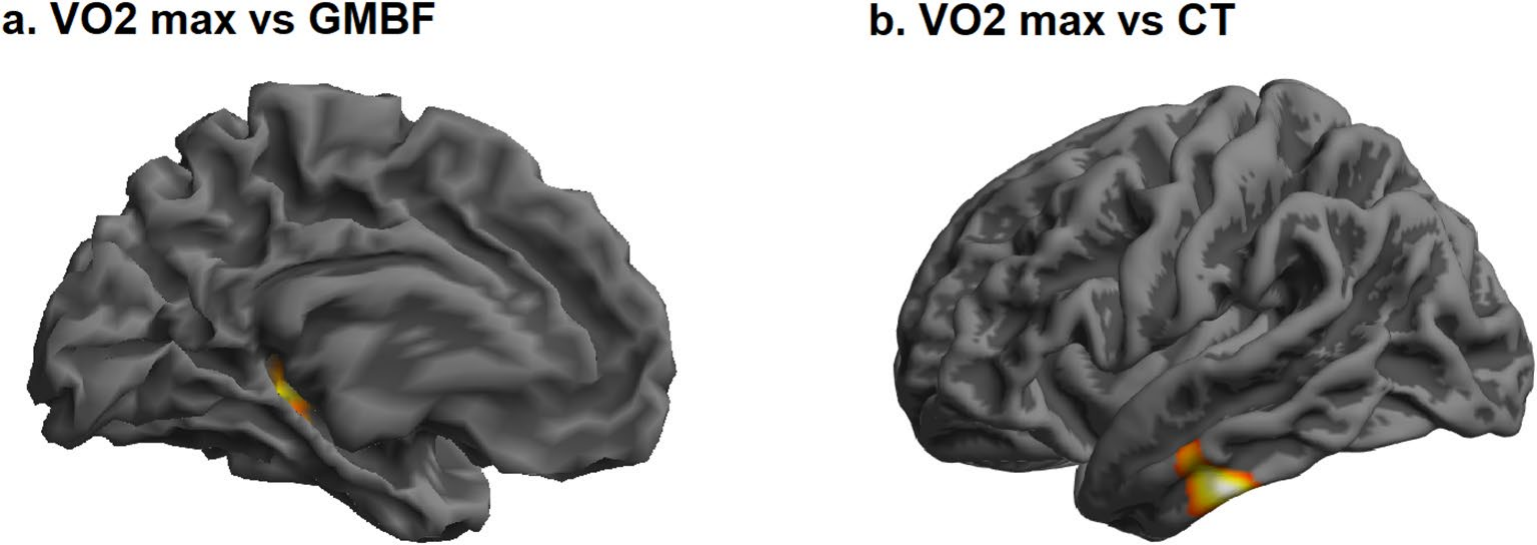
Association between VO_2_max and brain measures. The figure shows the clusters were a statistically significant association was detected between VO_2_max and (**a**) GMBF and (**b**) CT, respectively. (**a**) VO_2_max was negatively associated with GMBF of the left hippocampus (p FEW-corr = .0003, t = 5.64, MNI coordinates (x, y, z): −36, −34, 4). (**b**) VO_2_max was, on the other hand, positively associated with the CT of the left superior temporal gyrus (STG) (p FWE-corr = .006, t = 5.18, MNI coordinates (x, y, z): −56, −21, −3). Clusters were superimposed on a surface rendering in SPM12. The Harvard–Oxford Cortical Structural Atlas was used to derive the location of the coordinates.

In this voxel-wise exploratory analysis, a statistically significant negative correlation between VO_2_max and GMBF (p FWE-corr = 0.0003) was detected in the left hippocampus (179 voxels, t = 5.64, MNI coordinates (x, y, z): −36, −34, 4) (Fig. 3a).

#### Association between VO_2_max and grey matter volume

##### **H7**

VO_2_max will positively correlate with total grey matter volume

Linear regression was used to test for associations between VO_2_max (independent variable) and total GMV (dependent variable), corrected for age, gender and total intracranial volume (TIV) (GM + white matter + cerebrospinal fluid). No extreme outliers on GMV were detected. Homogeneity of variance was met at the NCV test (p = 0.979) and the residuals were normally distributed. The threshold for significance was set at p < 0.05.

No statistically significant associations were detected between VO_2_max and total GMV at either the uncorrected model (p = 0.745; CI 95%: −0.0012, 0.0008; r = 0.14; Cohen’s f = 0.14), nor after correcting for age, gender and TIV (r = 0.11; Cohen’s f = 0.11) (Table 2).

RQ4. Is VO_2_max related with regional-specific grey matter volume?

Linear regression was used to test for a correlation between VO_2_max (independent variable) and whole-brain, voxel-wise GMV (dependent variable), correcting for age, gender and TIV. The threshold for significance was set at p < 0.001; a further FWE-correction at p < 0.05 was applied to voxels surviving the preliminary uncorrected threshold^57^. Post-hoc analyses were masked for the main effect of VO_2_max.

No statistically significant associations were detected between VO_2_max and voxel-wise GMV.

#### Association between VO_2_max and cortical thickness

##### **H8**

VO_2_max will positively correlate with mean cortical thickness

Linear regression was used to assess associations between VO_2_max (independent variable) and mean CT dependent variable, correcting for age and gender. No extreme outliers on mean cortical thickness were detected. The NCV test was used to verify the homogeneity of variance (p = 0.893). Residuals were normally distributed. The threshold for significance was set at p < 0.05.

VO_2_max had a statistically significant positive association with mean CT both at the uncorrected model (p = 0.039; CI 95%: 0.001, 0.009) and after correction for age and gender, with medium effect sizes (r = 0.30 and 0.33, corresponding to Cohen’s f = 0.31 and 0.34, at the uncorrected and corrected model, respectively) (Table 2).

##### **RQ5**

Is VO_2_max related with regional-specific CT?

CAT-12 was used to test for a correlation between VO_2_max (independent variable) and whole-brain, vertex-wise CT (dependent variable). The analysis was corrected for age and gender. The threshold for significance was set at p < 0.001; a further FWE-correction at p < 0.05 was applied to voxels surviving the preliminary uncorrected threshold^57^. Post-hoc analyses were masked for the main effect of VO_2_max.

In the vertex-wise analysis, VO_2_max had a statistically significant positive association with the CT in the left superior temporal gyrus (STG) (p FWE-corr = 0.006, 181 voxels, t = 5.18, MNI coordinates (x, y, z): −56, −21, −3) (Fig. 3b).

## Discussion

We investigated the effects of moderate intensity physical exercise, relative to rest, on brain functional and structural measures, in older adults. A single session of moderate intensity physical exercise did not lead to appreciable changes in working memory performance, total GMV, CT or brain activity. However, mean CT was reduced at post-test independent of group, while regional CT was increased in the visual cortex. Cardiovascular fitness, measured as VO_2_max, was inversely associated with hippocampal blood flow and showed a trend for an inverse association with mean GMBF, in contrast with our hypotheses of a positive relation between VO_2_max and GMBF. On the other hand, VO_2_max and mean CT were positively associated in accordance with our hypothesis, and a positive association between VO_2_max and CT in the left STG was detected.

### Brain morphological measures: effect of exercise and association with VO_2_max

We report positive associations of VO_2_max with mean CT as well as with CT of the left STG. The left STG has largely been implicated in speech processing and comprehension^61,62^ and in speech-related social cognition^61,62^; however, in recent years its role in social cognition and information processing has also been highlighted^63–65^. Although few studies focused on CT measures in relation to aerobic fitness, previous research has reported specific positive associations of VO_2_max with the GMV^43,66^ and CT of the left STG^13,67^. Moreover, moderate-intensity aerobic training may slow down cortical thinning in several brain areas in patients with Alzheimer’s Disease^68^, and mean CT is also positively related with physical activity habits in cognitively healthy individuals^69^. The beneficial effect of cardiorespiratory fitness on body composition, and specifically body fat reduction, has been proposed to mediate the positive relationship between fitness and mean CT, at least in developing children^70^. In fact, adiposity is related with thinner cortex during development^70^. In older adults, cardiovascular fitness is however more likely to counteract the detrimental effects of aging on brain health by promoting neurons’ preservation and metabolism^2^.

Interestingly, the association between VO_2_max and brain morphology was specific to CT, while total GMV did not show any statistically significant association with VO_2_max. CT and GMV measurements are related; however, GMV measurements also depend on cortical folding and surface area^71–74^, and are more closely related with the latter rather than with CT^72^. CT and surface area are in fact genetically and phenotypically independent^72^, making GMV a composite^71,73,74^ and thus less readily interpretable measure of brain morphology^72^. Interestingly, CT seems to be more sensitive to atrophy compared with GMV^73,74^, an aspect to be considered when focusing on older age. This may also explain why we could not detect any effect of exercise or session on GMV.

Mean CT was also reduced at post-test independent of group. CT reductions may be associated with pruning of superfluous or weak synapses and increased myelination, which may occur already over minutes during fast learning^75^, speeding up the learning process by eliminating synapses producing high-error spikes^75^. We did observe changes in working memory performance; thus, it is possible that synaptic pruning may be partially contributing to the general decrease in mean CT at post-test. However, it is worth noting that mean GMBF was reduced at post-test in our sample^44^, which may be a potential cause for apparent reductions in mean CT^30^. Arterial blood has in fact larger T1 (relaxation time) than brain tissues, and perfusion changes can induce signal changes in T1-weighted images^30^. Physical exercise initially induces transient increases in cerebral blood flow^16,76^, independent of the intensity of exertion. The post-exercise hypotension and consequential drop in cardiac output, coupled with sympathetically-mediated vasoconstriction reflexes aimed at protecting the brain from the damage of hyper-perfusion, then leads to a quick decrease in cerebral blood flow, detectable a few seconds after the cessation of the exercise bout^16,76^. We did not find a direct relationship between cortical thickness and GMBF, and reduced mean GMBF was only observed in the exercise group^44^ rather than independently of group as was the case for mean CT; however, pulse pressure was increased in our sample after the first scanning session^44^ regardless of the intervention, possibly reflecting stress in relation to the experimental procedure^77^. Thus, we cannot rule out that changes in mean GMBF may have confounded our results.

The finding of increased CT in the visual cortex is, on the other hand, in line with previous reports of early increases in visual CT occurring already over very short periods of visual stimulation^27^, such as that elicited in our sample by the working memory task performed prior to the structural acquisition. Previous studies have suggested that such rapid changes in brain morphology may be ascribed to dendritic spine plasticity in the visual cortex^78^, or to changes in blood flow^27^. However, correcting our analysis for GMBF in the visual cortex did not alter our results, and GMBF and CT of the visual cortex did not correlate, suggesting that GMBF may not be the sole contributor to the increased CT in the primary visual cortex. Further research is warranted to disentangle the nature of such signal changes. However, the notion that T1-weighted signal can alter so rapidly is crucial to MRI research. Global CT measurements likely display sufficient measurement stability to detect subtle changes that may occur over short time scales as a consequence of undetermined physiological processes. As such, strict protocol control is necessary to ensure reproducibility within longitudinal studies as well as across studies.

Summarizing, we expanded current research on aerobic fitness by using a more precise measure such as VO_2_max, and by investigating different measures of grey matter morphology. In particular, we report positive associations of VO_2_max with CT, a measure often overlooked in favour of GMV despite its indication as a more sensitive measure of grey matter modifications in older age compared with GMV. We also support recent reports on the occurrence of rapid changes in CT in response to visual stimuli, extending previous findings to an older population. The nature of such changes is still debated. In this context, we support recent views that variations in blood flow may not be the sole contributor to rapidly detectable alterations in CT measurements. Although we cannot assert with absolute certainty that true plasticity may be at play, we nonetheless suggest that CT measurements may be sensitive to subtle undetermined physiological changes occurring over short time scales.

### Association between GMBF and VO_2_max

VO_2_max and cardiovascular fitness have been associated with higher middle cerebral artery compliance^38,39^ and higher cerebral blood flow in clinical^40^ and non-clinical populations^34,36,79^. Our study, however, supports more recent findings of negative associations between VO_2_max and resting GMBF^38,43^, specifically in the left hippocampus. Several mechanisms have been proposed to underlie discrepancies across studies. For example, differences in the VO_2_max range and cardiovascular health of the participants^43^, along with genetic variants potentially influencing brain perfusion, such as APoE4 variants associated with cerebral hyper-perfusion^43^, may all contribute to these somewhat conflicting literature findings. A recent hypothesis proposed by Furby et al.^38^ considers that fitter individuals may have more efficient gas exchange from the capillary bed due to increased vessel surface. This may lead to shorter diffusion distances and facilitate nutrient extraction, potentially reducing the amount of GMBF needed to meet metabolic oxygen demand^38^ and resulting in a negative association between GMBF and VO_2_max. Numerous preclinical studies have in fact demonstrated that regular physical exercise and aerobic fitness can increase brain vessel density^80–82^, also in the hippocampus^80,83^.

### Brain activity during the working memory task: effects of exercise and association with VO_2_max

We did not find any effect of a single session of physical exercise on working memory-related brain activity, contrary to previous studies. However, most of these studies have focused on children^32^, adolescents^31^, or young adults^20,25^. The only study carried out on older participants reported increased brain activation in the IFG and IPL and decreased activation in the anterior cingulate cortex after acute exercise compared with rest, during a conflict monitoring task^24^. Whether the effects might depend on the type of task and the executive sub-domain targeted^53,54^, or whether confounding factors such as exercise intensity might be at play, remains to be elucidated.

We did not detect any association of VO_2_max with working memory accuracy on any load. While similar findings of no associations between cardiovascular fitness and working memory have been previously reported in middle-aged Swedish office workers^84^, several studies have pointed toward a positive association between VO_2_max and working memory performance, at least in healthy adolescents and younger adults^85–88^, and in older adults with mild to moderate cognitive impairment^89^. The absence of clinically evident cognitive impairment in our sample, the level and variability in their fitness levels, and the small sample size may all have contributed to this discrepancy. Indeed, n-back back accuracy data were missing on nine participants, further limiting our power to detect small effects in correlation analyses and preventing us from stratifying the participants according to their fitness level, an important confounder when investigating cognitive measures^84^.

### Limitations and conclusions

This study is based on data from a project aiming at outlining the effect of acute physical exercise on the brain and cognition in older adults, and follows previous work focusing on cognitive performance and cerebral blood flow^44^. The sample size was somewhat small, limiting our power to detect small effects. Sample size was determined by statistical power analyses (G*Power), as reported in the preregistration (https://osf.io/m62rp/wiki/Power%20analysis/). However, n-back accuracy data were missing on nine participants, and seven subjects had to be excluded from our fMRI analysis due to movement, reducing our power in corresponding analyses. This might have contributed to the Moreover, we only assessed brain morphology twice, at pre- and post-test. Therefore, we cannot define the time-course and duration of the CT changes we observed, nor whether the effect of physical activity on brain morphology may be transient or sustained. While the mechanisms underlying those effects may be relatively transient, if repeated during habitual exercise, they may still induce sustained effects. Therefore, transient effects may be upstream causes of sustained effects. Future studies may be designed to acquire follow-up measurements of cerebral blood flow, brain morphology and cognition days or even weeks apart, to assess the time-course of brain changes induced by a single, acute bout of exercise. Such studies may also consider collecting device-based measures of physical activity and heart rate in the 24 h period prior to the acute exercise session.

Despite these limitations, we can conclude that a single session of moderate intensity exercise did not lead to appreciable changes in brain activity, working memory performance or total GMV in older individuals, compared to rest. VO_2_max, was associated with higher mean CT and higher CT in the left STG, but not with GMV. This supports the proposition that cardiovascular fitness is related with brain health in aging, and suggests that CT may be a more sensitive measure in older age compared with GMV. Furthermore, a negative association between VO_2_max and GMBF, particularly in the left hippocampus, was detected, reinforcing recent theories holding that fitter individuals may require lower GMBF levels to meet metabolic oxygen demand due to increased vessel surface and subsequent better capacity for gas exchange and nutrient extraction. Worth noting, associations between VO_2_max and imaging measures were limited to small, focal clusters. This may possibly indicate a lack of power to detect more widespread correlations, which might have been expected due to the global effect of aerobic activity and physical activity on brain circulation and morphology^3–7,11,34,36,37^.

We also support recent reports on the occurrence of rapid changes in CT in response to visual stimuli, extending previous findings to an older population. Regardless of the nature of such signal changes, the notion that T1-weighted signal can alter so rapidly is crucial to MRI research, hindering the reproducibility across studies using both structural and task-fMRI in the same session.

## Supplementary Information

Supplementary Information.

## Data Availability

Data are stored in compliance with the GDPR regulations. Data can be requested to the authors.
