# Supplementary material for: Higher VO2max is associated with thicker cortex and lower grey matter blood flow in older adults
Source: Sci Rep. 2021 Aug 18;11:16724. doi: 10.1038/s41598-021-96138-5 (PMC8373929; doi:10.1038/s41598-021-96138-5)
Supplement: Supplementary file 1 — Supplementary Information. [file 41598_2021_96138_MOESM1_ESM.docx]

**Supplementary Materials**

**Title:**

Higher VO_2_max is associated with thicker cortex and lower grey matter blood flow in older adults

**Authors:**

Gaia Olivo^1,2^, Jonna Nilsson^2,3^, Benjamín Garzón^1,2^, Alexander Lebedev^2,4^, Anders Wåhlin^5,6^, Olga Tarassova^3^, Maria M Ekblom^3,7^, Martin Lövdén^1,2^

*^1^ Department of Psychology, University of Gothenburg, Gothenburg, Sweden*

*^2^ Aging Research Center (ARC), Department of Neurobiology, Care Sciences and Society (NVS), Karolinska Institutet, Stockholm, Sweden*

*^3^ The Swedish School of Sport and Health Sciences, Stockholm, Sweden*

*^4^ Department of Clinical Neuroscience, Karolinska Institutet, Stockholm, Sweden*

*^5^* *Department of Radiation Sciences, Umeå University, Umeå, Sweden*

*^6^ Umeå Center for Functional Brain Imaging (UFBI), Umeå University, Umeå, Sweden*

*^7^ Department of Neuroscience, Karolinska Institutet, Stockhom, Sweden*

**Declarations of interest: none.**

**Corresponding author:**

Gaia Olivo, M.D., Ph.D.

Gaia.olivo@gu.se

Department of Psychology

University of Gothenburg

Haraldsgatan 1, 413 14 Göteborg, Sweden

**APPENDIX A. Pre-processing of imaging data**

*fMRI preprocessing for BOLD signal analyses*

Statistical Parametric Mapping 12 (SPM 12) and DPARSFA (http://rfmri.org/DPARSF) were used for the preprocessing of fMRI data. Functional images were slice-timing corrected and realigned to correct for head motion. Seven subjects had moved more than 3 mm in at least one session, and were thus excluded from the analysis. For each subject, the structural image was coregistered to the functional series. Structural images were segmented into grey matter, white matter and cerebrospinal fluid probability maps. DARTEL ^2^ was used to create a sample specific template. Structural images were normalized to the MNI space. The deformation parameters deriving from this procedure were applied to the corresponding functional images to normalize them to the MNI space ^3^. The images were then resampled to a 3 mm^3^ voxel-size and smoothed with an 8 mm FWHM Gaussian kernel. The 1-back task was used as baseline condition, given the less demanding cognitive load ^4^. First level (individual level) analyses were carried on each participant to calculate the following contrasts: 2-back vs 1-back; 3-back vs 1-back. Motion parameters were included as nuisance regressors.

*Voxel-based morphometry (VBM): longitudinal pipeline*

The longitudinal pipeline implemented in CAT 12 (http://www.neuro.uni-jena.de/cat/) was used for pre-processing. Briefly, this consists in an initial inverse-consistent rigid registration including a bias-correction between time points. The realigned images of all time points are then segmented into grey matter (GM), white matter (WM) and cerebrospinal fluid (CSF) probability maps. Registration parameters are estimated using a non-linear spatial registration and then averaged; the mean deformation parameters are then applied to segmented tissue maps and modulated. Smoothing with a 8 mm FWHM Gaussian kernel was applied. The Mahalanobis distance between mean correlation (a measure of the homogeneity of the pre-processed data) and weighted overall image quality (a measure of noise and spatial resolution of the original images) was used to assess overall data quality. Seven data-points were flagged based on this measure; however, as pointed out by CAT 12 developers, there is no clear cut-off to exclude a volume based on this measure, which should only be intended as a warning to proceed to careful visual inspection of the flagged data (http://www.neuro.uni-jena.de/cat12/CAT12-Manual.pdf). Only two of the flagged data-points presented with severe ring artefacts at visual inspection and were thus excluded from further analysis. Both subjects belonged to the resting group. Total GM volume was calculated from the non-modulated image of the remaining subjects.

*Voxel-based morphometry (VBM): cross-sectional pipeline*

Structural data were pre-processed with CAT12. All images were visually inspected for the presence of artefacts prior to pre-processing. The images were segmented into grey matter, white matter and cerebrospinal fluid probability maps. The probability maps were then normalized to the MNI space, resampled, and modulated with the Jacobian determinants to preserve information about their original volumes. A quality check was performed by plotting the Mahalanobis distance between mean correlation (a measure of the homogeneity of the pre-processed data) and weighted overall image quality (a measure of noise and spatial resolution of the original images). Grey matter maps were smoothed with 8 mm FWHM Gaussian kernel.

Cortical thickness and surface reconstruction were also obtained as part of the CAT12 pipeline with a projection-based thickness (PBT) approach. According to the PBT approach, tissue segmentation is used to estimate the distance between white matter distance and grey matter surface, then local maxima (corresponding to the cortical thickness) are projected onto other GM voxels using a neighbouring algorithm depending on the white matter distance from grey matter surface. Surface images were smoothed with a 12 mm FWHM Gaussian kernel prior to statistical analyses.

*Cortical thickness analysis*

Cortical thickness and surface reconstruction were obtained from CAT 12 as part of the longitudinal pipeline. A projection-based thickness (PBT) approach is implemented as part of this pipeline. Briefly, tissue segmentation is used to estimate the white matter (WM) distance, then local maxima (corresponding to the cortical thickness) are projected onto other GM voxels using a neighbouring algorithm depending on the white matter distance. The PBT allows the handling of partial volume information, sulcal blurring, and sulcal asymmetries. Surface images were then smoothed with a 12 mm FWHM Gaussian kernel.

*Preprocessing of structural images for the arterial spin labelling analysis*

The pre-processing of the structural images to be fed into the BASIL pipeline followed the fsl_anat pipeline, consisting of the following steps: (1) reorienting the images to match the standard Montreal Neurological Institute (MNI) space; (2) the images are then cropped and (3) bias-field correction is applied, using the FAST (FMRIB's Automated Segmentation Tool) algorithm ^5^; (4) linear and non-linear registration of the images to the MNI space is achieved using, respectively, FLIRT (FMRIB's Linear Image Registration Tool) ^6^ and FNIRT (FMRIB's Non-linear Image Registration Tool); (5) brain extraction is performed with the Bet Extraction Toolbox (BET) ^7^ and (6) tissue-type segmentation is obtained using the FAST algorithm for cortical structures and the FIRST (FMRIB’s Integrated Registration and Segmentation Tool) for subcortical structures.

**APPENDIX B. GRAPH THEORY (MODULARITY) ANALYSIS.**

*Rationale for the selection of modularity as network measure*

FMRI provides an indirect measure of brain activity based on the blood–oxygen‐level‐dependent (BOLD) signal, which is sensitive to cerebral blood flow changes occurring in response to task-related metabolic demands (i.e., the neurovascular coupling). Therefore, individual differences in the neurovascular coupling (e.g. in aging) may confound this signal ^8^. . Such confounds are, however, less concerning when graph theory approaches are used ^8^.

Connectivity-based analyses describe the associations between BOLD response in different brain areas. Graph theoretical analysis of such associations can be used to describe the topological and architectural structure of functional brain networks ^9^. The brain comprises a set of networks with a complex cross-talk between each other. These brain networks can be characterized with several metrics. Amongst them, the modularity of brain networks has recently become particularly salient ^9^. Modularity measures the extent to which brain networks can be subdivided (i.e. segregated) into distinct subnetworks, called modules. Modules are represented by a collection of regions, called nodes, typically having many connections with other nodes within their own subnetwork and fewer connections with nodes belonging to other subnetworks ^10^. Modularity measures how “segregated” the subnetworks are. High modularity indicates highly segregated subnetworks, featuring many within-module connections and very few between-modules connections. Low modularity indicates poorly segregated subnetworks, with high between-modules cross-talk ^10^.

Modularity has been suggested as a useful marker of the plasticity of cognitive performance ^10^, particularly of intervention-related changes in performance induced by repeated sessions of cognitive training or aerobic exercise ^10^. Individual differences in pre-intervention modularity predict gains in cognitive performance across healthy and clinical adult populations in both these intervention settings ^10^. Moreover, higher VO_2_max has been linked with higher brain network modularity ^11^. Higher brain network modularity, reflective of greater segregation between brain networks, is in turn associated with better WM performance in older adults ^12^, and relates to larger gains in executive function after 6 months of aerobic exercise intervention in older adults ^13^, and to better WM performance ^12,14^.

*fMRI preprocessing for modularity analysis*

For modularity analysis, further preprocessing steps were carried out. A band-pass filter of 0.01-0.1 Hz was applied to the functional images to remove residual motion and physiological artefactual effects from the BOLD signal; additionally, the noise due to white matter, cerebrospinal fluid, and motion were regressed out. The CompCor method, based on the identification of voxel-wise principal components from anatomical-based regions of interest, was used for the removal of white matter and CSF signal from the data ^15^. Frames with DVARS values exceeding the threshold of 75th percentile + 1.5 times the InterQuartile Range (IQR), as calculated with the fsl_motion_outliers command in FSL (FMRIB Software Library) ^16^ were excluded. Functional maps were parcellated into 264 regions, according to the Power et al. 2011 ^17^ parcellation scheme. Blocks on each load were concatenated ^18^. Brain network modularity was calculated separately for each load using the Brain Connectivity Toolbox (BCT) ^19^, by applying the Louvain’s algorithm. Prior to the computation, NaN values were removed and self-connections weights were set to 0. The asymmetric treatment of negative weights was chosen for the computation, to account for the unequal importance of positive and negative weights in modularity-partition determination ^20^. Contrary to positive weights, which associated nodes with modules explicitly, negative weights in fact do rather associate nodes with modules by dissociating them from other modules ^20^.

Whole-brain modularity was calculated. As reported in the preregistration, we planned to not apply any global signal regression to avoid removing task-relevant signal variance ^12^ in the main analysis, but to report the results after applying GSR as well to account for the lack of consensus concerning the regression of global signal from fMRI data ^21^. However, whole-brain modularity values calculated prior to GSR were very low. We therefore decided to report the analysis with GSR in the main manuscript and report the results without GSR as supplementary material. The correlation between modularity calculated with and without GSR is depicted in figure S1.

**Figure S1**

**
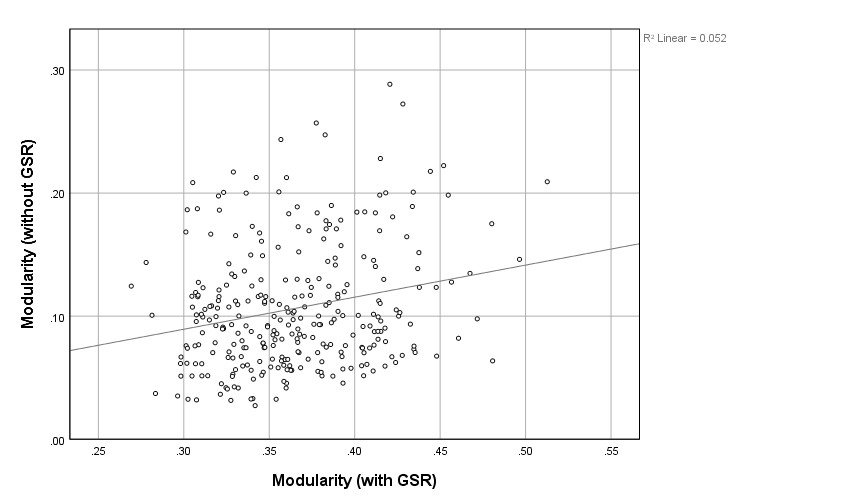
**

**Figure S1. Correlation between modularity values calculated with and without applying global signal regression.** Modularity values calculated with and without global signal regression show a statistically significant correlation; however, the correlation was low (Spearman’s Rho=.192).

*Statistical analysis*

According to the preregistration, Brain Connectivity Toolbox (BCT) should have been used to analyse task-related brain network modularity. However, given the limited statistical modelling available within the toolbox, modularity values relative to each load of the WM task were instead extracted and imported in SPSS for the statistical analyses. Modularity was not normally distributed at the Shapiro Wilk’s test for normality, and was right-skewed. No extreme outliers (values that lie more than three times the IQR below the first quartile or above the third quartile) were found for any group combination (group*session*load). A generalized linear mixed model with a gamma distribution with log link was used for the analysis. Group was set as between-subject factor; session and load represented the within-subject factors. The subject factor was entered as random factor in the model. The threshold for significance was set at p < .05. To ensure that misunderstanding of the instructions and/or the excessive difficulty on the higher loads were not confounding the results, the same analysis was also repeated after removing the moderate outliers on the behavioural performance. The accuracy of the retained individuals was above .94 on the 1-back task, above .65 on the 2-back task and above .59 on the 3-back task.

*Results*

Pre- to post-test modularity measures were correlated in both the exercise (Spearman’s Rho = .297,p = .011) and rest group (Spearman´s Rho = .316, p = .006) across loads. No statistically significant effects of group, session, load, or their interactions on whole-brain modularity were found, neither when applying GSR (figure S2; table S1) nor without it (table S2). However, a significant session*load effect was found after GSR (F_(2,82)_=3.798; p = .024), indicating increased modularity at post-test on the 1-back task only (Contrast Estimate=.016; Confidence Intervals 95% (min, max)=.003, .028; p = .014; Cohen´s d=.349, medium effect size). After removing the moderate outliers on behavioural performance, the results were unchanged (not reported).

**Figure S2**

*
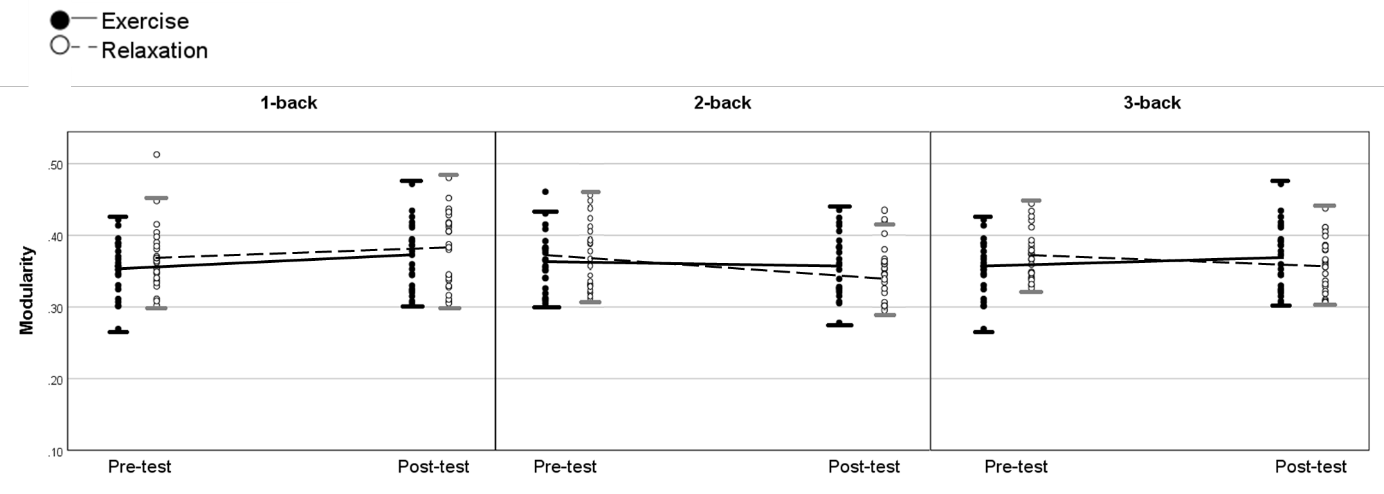
*

**Figure S2. Whole-brain modularity as a function of session, load, and group.** The figure shows the pre- to post-test change in brain modularity in the exercise (black line) and rest (dotted line) groups relative to the 1-back (left), 2-back (middle) and 3-back (right) tasks, after applying GSR. Individual data (black circles for the exercise group; white circles for the rest group) are also plotted, and interquartile range (IQR) are reported (±1.5 IQR). No statistically significant group*session or group*session*load effects were detected; however, a statistically significant session*load interaction was found, indicating increased modularity at post-test on the 1-back, independent of group. Mean modularity of the whole sample was .36 (sd .04).

**Table S1. Effects of group, session and load on modularity after global signal regression (n=49).**

|  | **C.E.** | **S.E.** | **t** | **Adj. Sig.** | **95% C.I. (min, max)** |
| --- | --- | --- | --- | --- | --- |
| *Group, F(1,282)=.231; p=.631* |  |  |  |  |  |
| Exercise vs Resting | -.004 | .008 | -.481 | .631 | -.019, .012 |
|  | | | | | |
| *Session, F(1,282)=.107; p=.744* | | | | | |
| Post-test vs Pre-test | .001 | .004 | .327 | .744 | -.007, .009 |
|  | | | | | |
| *Load, F(2,282)=1.258; p=.286* | | | | | |
| 1- vs 2-back | .008 | .005 | 1.550 | .122 | -.002, .018 |
| 1- vs 3-back | .002 | .005 | .423 | .672 | -.008, .013 |
| 2- vs 3-back | -.006 | .005 | -1.038 | .300 | -.016, .005 |
|  | | | | | |
| *Group*session, F(1,282)=2.452, p=.119* | | | | | |
| Pre-test, Exercise vs Resting | -.010 | .009 | -1.182 | .238 | -.027, .007 |
| Post-test, Exercise vs Resting | .003 | .009 | .278 | .781 | -.016, .021 |
|  | | | | | |
| *Group*load, F(2,282)=1.171; p=.312* | | | | | |
| 1-back, Exercise vs Resting | -.012 | .011 | -1.153 | .250 | -.034, .009 |
| 2-back, Exercise vs Resting | .002 | .009 | .230 | .818 | -.016, .021 |
| 3-back, Exercise vs Resting | -.001 | .009 | -.128 | .898 | -.020, .017 |
|  | | | | | |
| **† *Session*load, F(2,282)=3.798; p=.024*** | | | | | |
| **1-back, Post-test vs Pre-test †** | **.016** | **.006** | **2.482** | **.014** | **.003, .028** |
| 2-back, Post-test vs Pre-test | -.009 | .007 | -1.287 | .199 | -.022, .005 |
| 3-back, Post-test vs Pre-test | -.003 | .008 | -.330 | .742 | -.018, .013 |
|  | | | | | |
| *Group*session*load, F(2,282)=.446; p=.640* | | | | | |
| Pre-test, 1-back, Exercise vs Resting | -.015 | .011 | -1.279 | .202 | -.037, .008 |
| Pre-test, 2-back, Exercise vs Resting | -.003 | .012 | -.241 | .810 | -.026, .020 |
| Pre-test, 3-back, Exercise vs Resting | -.013 | .012 | -1.089 | .277 | -.036, .010 |
| Post-test, 1-back, Exercise vs Resting | -.010 | .014 | -.756 | .450 | -.037, .016 |
| Post-test, 2-back, Exercise vs Resting | .007 | .012 | .612 | .541 | -.016, .030 |
| Post-test, 3-back, Exercise vs Resting | .010 | .013 | -.817 | .414 | -.015, .035 |
|  | | | | | |
| *C.E., contrast estimate; S.E., standard error; df, degrees of freedom; C.I., confidence intervals.* | | | | | |

**Table S2. Effects of group, session and load on modularity without global signal regression (n=49).**

|  | **C.E.** | **S.E.** | **t** | **Adj. Sig.** | **95% C.I.**  **(min, max)** |
| --- | --- | --- | --- | --- | --- |
| *Group, F(1,282)=2.151; p=.144* | |  |  |  |  |
| Exercise vs Resting | .014 | .009 | 1.482 | .139 | -.004, .032 |
|  |  |  |  |  |  |
| *Session, F(1,282)=.003; p=.956* |  |  |  |  |  |
| Post-test vs Pre-test | <.001 | .004 | .055 | .956 | -.008, .008 |
|  |  |  |  |  |  |
| *Load, F(2,282)=.192; p=.825* |  |  |  |  |  |
| 1- vs 2-back | .003 | .005 | .526 | .600 | -.007, .012 |
| 1- vs 3-back | <.001 | .005 | .047 | .963 | -.010, .011 |
| 2- vs 3-back | -.002 | .005 | -.497 | .619 | -.011, .007 |
|  |  |  |  |  |  |
| *Group*session, F(1,282)=.250, p=.618* | | |  |  |  |
| Pre-test, Exercise vs Resting | .012 | .010 | 1.215 | .225 | -.007, .031 |
| Post-test, Exercise vs Resting | .016 | .010 | 1.499 | .135 | -.005, .036 |
|  |  |  |  |  |  |
| *Group*load, F(2,282)=2.326; p=.100* | |  |  |  |  |
| 1-back, Exercise vs Resting | .019 | .011 | 1.661 | .098 | -.004, .042 |
| 2-back, Exercise vs Resting **†** | .020 | .010 | 2.075 | .039 | .001, .040 |
| 3-back, Exercise vs Resting | .002 | .011 | .137 | .891 | -.021, .024 |
|  |  |  |  |  |  |
| *Session*load, F(2,282)=2.385 p=.094* | |  |  |  |  |
| 1-back, Post-test vs Pre-test **†** | .013 | .006 | 2.054 | .041 | .001, .025 |
| 2-back, Post-test vs Pre-test | -.006 | .008 | -.839 | .402 | -.021, .008 |
| 3-back, Post-test vs Pre-test | -.006 | .008 | -.718 | .473 | -.021, .010 |
|  |  |  |  |  |  |
| *Group*session*load, F(2,282)=.484; p=.617* | | |  |  |  |
| Pre-test, 1-back, Exercise vs Resting | .020 | .013 | 1.595 | .112 | -.005, .046 |
| Pre-test, 2-back, Exercise vs Resting | .013 | .012 | 1.156 | .249 | -.009, .036 |
| Pre-test, 3-back, Exercise vs Resting | <.001 | .014 | .030 | .976 | -.028, .029 |
| Post-test, 1-back, Exercise vs Resting | .017 | .013 | 1.300 | .195 | -.009, .044 |
| Post-test, 2-back, Exercise vs Resting **†** | .027 | .013 | 2.024 | .044 | .001, .053 |
| Post-test, 3-back, Exercise vs Resting | .003 | .013 | .199 | .843 | -.029, .024 |
| *C.E., contrast estimate; S.E., standard error; df, degrees of freedom; C.I., confidence intervals; †, statistically significant* | | | | | |

**APPENDIX C. ADDITIONAL PREREGISTERED HYPOTHESES.**

Additional hypotheses concerning the mediatory role of GMBF on brain measures changes were preregistered, as well as hypotheses on potential associations between WM performance and changes on brain measures. As no exercise-related changes on either WM performance, brain activity or GM measures were detected, these hypotheses were no longer valid. We have decided to report these hypotheses, as well as the corresponding statistical analyses and results, below.

*H1. Exercise-induced changes in cerebral blood flow will mediate voxel-wise task-related activity changes*

According to the preregistration, we intended to test whether exercise-related changes in brain activity during the working memory task would be mediated by underlying changes in local blood flow, by testing for group*session and group*session*load interactions with the addition of a voxel-based correction for cerebral blood flow. The analyses should have been masked for the clusters showing a significant group*session*load interaction at the previous analysis. Given the null findings at the previous analysis, however, running a biological parametric mapping analysis was deemed no longer appropriate.

*H2. Brain modularity will correlate with working memory performance more strongly in the exercise group compared with the resting group*

SPSS was used to test whether pre-test to post-test changes in brain network modularity were associated with pre-test to post-test changes in working memory performance, and whether between-groups differences existed in the correlation coefficients. Given the lack of detectable effects of group on both working memory performance ^25^ and modularity (see below) we no longer expected the group to moderate the association; we did nonetheless perform this analysis as exploratory. Pre-test to post-test changes on brain modularity and working memory accuracy were computed for each load separately. A generalized linear mixed model was used to test for an effect of the group*load*brain modularity changes interaction on the working memory accuracy change. The change on working memory accuracy was normally distributed in each load and group, thus a linear distribution was chosen. Two extreme outliers on the working memory performance, identified by previous analyses ^25^, were excluded from the analysis. Mild outliers were retained as they did not impact on previous analyses ^25^ and can be representative of the normal variability within the population when operating on small sample sizes. The threshold for significance was set at p < .05. Group-specific post-hoc tests were performed when appropriate.

*H3. Pre- to post-test changes in total GM volume will correlate with changes in GMBF more strongly in the exercise group*

The difference in total GM volume between sessions was computed (post-test vs pre-test). A positive difference reflected a post-test increase in total GM. One subject was an extreme outlier on the total GM difference and was excluded from the correlation analysis. Total GM volume difference was left-skewed (skewness: -1.083). Log transformation of the variable was not achievable due to the presence of negative values. The variable was thus reflected, according to the formula:

Xi_reflected = Xmax+1-Xi

Where Xmax represent the maximum value for the sample. The square root of the reflected variable was then calculated. The final transformed total GM volume showed a perfect inverse correlation with the original total GM difference (Spearman’s Rho = -1.000) and a slightly positive skewness (skewness: .273). At the Shapiro Wilk’s test for normality, however, the transformed GM difference was normally distributed in the exercise group (p=.366) but not in the resting group (p=.040). A generalized linear model with a gamma distribution was thus used, including group, GMBF, and GMBF*group effects. A robust estimation was chosen. The group*GMBF changes. The threshold for significance was set at p < .05.

*H4. Pre- to post-test changes in total GM volume would correlate with changes on n-back performance more strongly in the exercise group*

The difference in n-back accuracy (post-test versus pre-test) was computed. A positive difference reflected a post-test increase in accuracy. A generalized linear model with linear distribution and robust estimation was chosen. A full-factorial model was carried out to test for effects of group, total GM volume difference, and group*GM volume difference interaction on n-back difference in accuracy. The group*GM volume difference was the effect of interest. The threshold for significance was set at p < .05.

*H5. The exercise-induced increase in GMBF will be mediating the association between WM and total GM volume*

We had hypothesized that the exercise-induced pre- to post-change on total GM volume would correlate with the change on n-back accuracy, and that such relationship would be mediated by changes on GMBF. However, contrary to our hypotheses, we did not find any association between total GM volume and GMBF nor between GM volume and WM performance (see below). Thus, a mediation analysis was no longer appropriate or necessary, and was not performed.

*H6. Pre- to post-test changes in mean CT would correlate with changes on n-back performance more strongly in the exercise group*

The difference in n-back accuracy (post-test versus pre-test) was computed. A positive difference reflected a post-test increase in accuracy. A generalized linear model with linear distribution and robust estimation was chosen. A full-factorial model was carried out to test for effects of group, mean CT change, and group*CT change interaction on n-back difference in accuracy. The group*CT change was the effect of interest. The threshold for significance was set at p < .05.

*H7. The exercise-induced increase in GMBF will be mediating the association between WM and mean CT*

We had hypothesized that the exercise-induced pre- to post-change on mean CT would correlate with the change on n-back accuracy, and that such relationship would be mediated by changes on GMBF. However, contrary to our hypotheses, we did not find any association between mean CT and GMBF nor between mean CT and WM performance (see below). Thus, a mediation analysis was no longer appropriate or necessary, and was not performed.

*H8. VO_2_max will positively correlate with brain network modularity*

No extreme outliers on modularity were detected at any load. Homogeneity of variance was confirmed by the NCV test (1-back, p=.140; 2-back, .065; 3-back, p=.879). Residuals on the 1- and 2-back modularity did not follow a normal distribution; residuals on 3-back modularity were normally distributed. A log-transformation was applied to the data. After the log-transformation, the residuals on 2-back and 3-back modularity were normally distributed, while residuals on the 1-back modularity only approached normality of the distribution. Given that ANOVA is deemed to be quite robust to violations of the normality assumption ^26,27^, repeated-measures ANOVA was applied. A full-factorial model (VO2max, load, load*VO2max) was carried out, where VO_2_max and load*VO_2_max were the effects of interest. The analysis was corrected for age and gender. The threshold for significance was set at p <.05.

*RQ1. Changes on grey matter will be associated with changes in* *GMBF*

According to the preregistration, we intended to test whether exercise-related changes in voxel-wise grey matter volume would be mediated by underlying changes in local blood flow, by testing for the group*session interaction with the addition of a voxel-based correction for cerebral blood flow. Given the null findings at the VBM analysis, this hypothesis was no longer supported and the relative analysis not justified.

*RQ2. How do VO_2_max, brain variables, and WM relate to each other?*

Partial correlations of VO_2_max with WM performance, mean GMBF, brain network modularity, total GMV and mean CT were tested using Spearman’s coefficient. Pairwise correlations were assessed after partialing out the effect of all other variables and correcting for age and sex. The model included 37 subjects. Fractional GMV, corresponding to GMV/TIV, was used for this analysis. Given the exploratory nature of the analysis, a false-discovery rate (FDR) approach, less conservative than the Bonferroni approach, was used to correct for multiple testing with an alpha-level of .05.

*Results*

Pre- to post-test changes on total grey matter volume and mean cortical thickness did not correlate with changes in mean GMBF (table S3). No associations were found between pre- to post-test changes in working memory performance and pre- to post-changes in modularity, grey matter volume or cortical thickness (table S4). Baseline modularity did not predict working-memory gains, and no statistically significant group*modularity, load*modularity, or group*load*modularity interactions were found either. The results were unchanged when analysing modularity data without GSR.

No statistically significant effect of VO_2_max or load*VO_2_max interaction on brain modularity was found at the repeated-measure ANOVA, either at the uncorrected model nor when correcting for age and gender (table S5). When examining each load separately, VO_2_max was not significantly associated with modularity at any load (p > .399). When fully adjusting each pairwise correlation for the all the other variables (n=37), trends for correlations between 2-back accuracy and mean CT, and between 2-back accuracy and task-related modularity were detected, not surviving the correction for multiple testing.

**Table S3. Correlations between pre- to post-test difference on morphological measures and GMBF (n=49).**

|  | **Exp(B)** | **S.E.** | **Sig.** | **C.I. 95% (min, max)** |
| --- | --- | --- | --- | --- |
| Total grey matter volume |  |  |  |  |
| GMBF | .992 | .0165 | .623 | .960, 1.025 |
| GMBF, Exercise vs Resting | .967 | .0241 | .168 | .923, 1.014 |
| Mean cortical thickness |  |  |  |  |
| GMBF | 1.001 | .0008 | .138 | 1.000, 1.003 |
| GMBF, Exercise vs Resting | 1.000 | .0011 | .709 | .997, 1.002 |
| *S.E., standard error; df, degrees of freedom; C.I., confidence intervals; GMBF, grey matter blood flow.* | | | | |

**Table S4. Correlations between n-back performance and GM measures (n=40).**

|  | **Exp(B)** | **S.E.** | **Sig.** | **C.I. 95% (min, max)** |
| --- | --- | --- | --- | --- |
| Total grey matter volume |  |  |  |  |
| GM volume difference | 1.000 | .0004 | .631 | .999, 1.001 |
| GM, Exercise vs Resting | 1.000 | .0009 | .664 | .999, 1.002 |
| Mean cortical thickness |  |  |  |  |
| CT difference | 1.128 | .1067 | .260 | .915, 1.390 |
| CT, Exercise vs Resting | 1.001 | .3561 | .998 | .498, 2.011 |
| *S.E., standard error; df, degrees of freedom; C.I., confidence intervals; GM, grey matter; CT, cortical thickness*  **Table S5. Effect of VO_2_max on n-back accuracy and modularity (ANOVA).**   \|  \| **Df** \| **Sum Square** \| **Mean Square** \| **F** \| **P value** \| **Cohen’s f** \| \| --- \| --- \| --- \| --- \| --- \| --- \| --- \| \| ***Accuracy (n=40)*** \| \| \| \| \| \| \| \| \| LOAD \| 2 \| 1.093 \| .5465 \| 188.754 \| < .001 \| 1.86 \| \| VO_2_MAX \| 1 \| .0024 \| .0024 \| .836 \| .363 \| .09 \| \| LOAD * VO_2_MAX \| 2 \| .0056 \| .0028 \| .963 \| .385 \| .13 \| \| ***Modularity (n=49)*** \| \| \| \| \| \| \| \| \| LOAD \| 2 \| .0074 \| .0037 \| .283 \| .754 \| .06 \| \| VO_2_MAX \| 1 \| .0008 \| .0008 \| .059 \| .809 \| .02 \| \| LOAD * VO_2_MAX \| 2 \| .0073 \| .0036 \| .278 \| .758 \| .06 \|   *Corrected for age and gender. Df, degrees of freedom.* | | | | |

**Appendix D. Supplementary tables and figures.**

**Table S6. Sample characterization at pre-test.**

|  | **Exercise** | **Resting** |
| --- | --- | --- |
|  | **(mean (SD))** | **(mean (SD))** |
| *N* | 24 | 25 |
| *Age (years)* | 69.6 (2.8) | 70.7 (3.1) |
| *VO2 max (ml/min/kg)* | 31.4 (5.5) | 32.3 (5.6) |
| *MMMSE* | 28.9 (1.2) | 28.4 (1.0) |
| *IPAQ (MET/min/week)* | 2731.0 (1442.0) | 3268.2 (2027.) |
| *Systolic blood pressure (mmHg)* | 136.1 (16.4) | 140.8 (16.7) |
| *Diastolic blood pressure (mmHg)* | 87.8 (11.1) | 86.5 (9.7) |
| *Memory evaluation* | 3.6 (0.7) | 3.6 (0.7) |
| *Memory vs 20 years old* | 3.1 (0.9) | 3.4 (0.7) |
| *Plasticity belief* | 2.1 (0.9) | 2. (1.2) |
|  | **%** | **%** |
| *Sex (females; males)* | 50.0; 50.0 | 40.0; 60.0 |
| *Marital status (married)* | 66.7 | 56 |

*SD, standard deviation*

**Table S7. Effect of the group on total GM volume (n=49).**

|  | **C.E.** | **S.E.** | **t** | **Adj. Sig.** | **C.I. 95% (min, max)** |
| --- | --- | --- | --- | --- | --- |
| *Group, F(1,90)=..055; p=.815* | | | | | |
| Exercise vs Resting | -3.57 | 15.169 | -.235 | .814 | -33.707, 26.566 |
| *Session, F(1,90)=3.453; p=.066* | | | | | |
| Post-test vs Pre-test | -1.989 | 1.067 | -1.864 | .066 | -4.109, .131 |
| *Group*session F(1,90)=.316; p=.576* | | | | | |
| Exercise, post- vs pre-test | -1.383 | 1.203 | -1.150 | .253 | -3.774, 1.07 |
| Resting, post- vs pre-test | -2.597 | 1.767 | -1.470 | .145 | -6.108, .914 |
| C.E., contrast estimate; S.E., standard error; df, degrees of freedom; C.I., confidence intervals. | | | | | |

**Table S8. Effect of load on brain activity during n-back performance (n=42).**

|  |  |  | **MNI** | | |  |
| --- | --- | --- | --- | --- | --- | --- |
| **kE** | **P FWE** | **T** | **x** | **y** | **z** | **Structure** |
| *2-back > 1-back* | | | | | | |
| 9550 | < .001 | 11.21 | -36 | -54 | 45 | L Superior Parietal lobule, Angular Gyrus, Supramarginal Gyrus |
|  |  | 11.08 | -27 | -75 | 39 | L Superior lateral occipital cortex |
|  |  | 11.01 | -9 | -59 | 64 | L Precuneus |
| 6946 | < .001 | 10.85 | -45 | 21 | 30 | L Middle frontal gyrus |
|  |  | 10.11 | -30 | 21 | -3 | L Insula |
|  |  | 10.09 | 9 | 21 | 42 | R Paracingulate gyrus |
| *3-back > 1-back* | | | | | | |
| 5528 | < .001 | 12.85 | -42 | 18 | 30 | L Middle frontal gyrus |
|  |  | 11.09 | -24 | 6 | 51 | L Superior frontal gyrus |
|  |  | 10.59 | 27 | 3 | 51 | L Middle frontal gyrus |
| 6124 | < .001 | 11.56 | -27 | -78 | 39 | L Superior lateral occipital cortex |
|  |  | 11.56 | -24 | -63 | 45 | L Superior lateral occipital cortex |
|  |  | 11.55 | -30 | -72 | 33 | L Superior lateral occipital cortex |
| 249 | .001 | 7.88 | 33 | 24 | 3 | R Insula |
| *1-back > 2-back* | | | | | | |
| 233 | .004 | 6.55 | -51 | 9 | 6 | L Inferior frontal gyrus |
| 124 | .043 | 5.37 | 39 | 9 | -33 | R Temporal pole |
| 367 | < .001 | 5.37 | 42 | -6 | 6 | R Insula |
| 119 | .048 | 5.08 | 3 | 33 | -12 | R Paracingulate gyrus, medial frontal cortex |
| *1-back > 3-back* | | | | | | |
| 861 | < .001 | 8.32 | -6 | 60 | 3 | L Frontal pole |
| 985 | < .001 | 6.97 | 60 | -24 | 24 | R Supramarginal gyrus |
| 147 | .014 | 5.98 | -36 | -15 | 69 | L Precentral gyrus |
| 659 | < .001 | 5.98 | -51 | -6 | 9 | L Central Operculum |
| 321 | < .001 | 5.92 | -3 | -9 | 48 | L Supplementary motor cortex |
| 166 | .009 | 5.91 | -3 | -48 | 30 | L Posterior cingulate cortex |
| *2-back > 3-back* | | | | | | |
| 156 | .013 | 5.04 | 24 | -51 | -18 | R lobule VI |
| 114 | .039 | 5.02 | 3 | -18 | 39 | R Posterior cingulate cortex |
| 182 | .007 | 5.01 | 45 | 27 | -12 | R Orbitofrontal cortex |
| 248 | .002 | 4.72 | 60 | -45 | 30 | R Supramaginal gyrus |
| 378 | < .001 | 4.67 | 3 | -45 | 15 | R Posterior cingulate cortex |
| 405 | < .001 | 4.62 | 6 | 18 | 63 | R Superior Frontal Gyrus |
| 141 | .019 | 4.54 | -45 | 24 | -12 | L Orbitofrontal cortex |
| 145 | .017 | 4.45 | -21 | -84 | -27 | L Crus I |
| 494 | .002 | 20.5 | 4 | -80 | -12 | R Lingual gyrus |

*kE, cluster extent (voxels); FWE, family-wise error; R, right; L, left.*

**Figure S3**


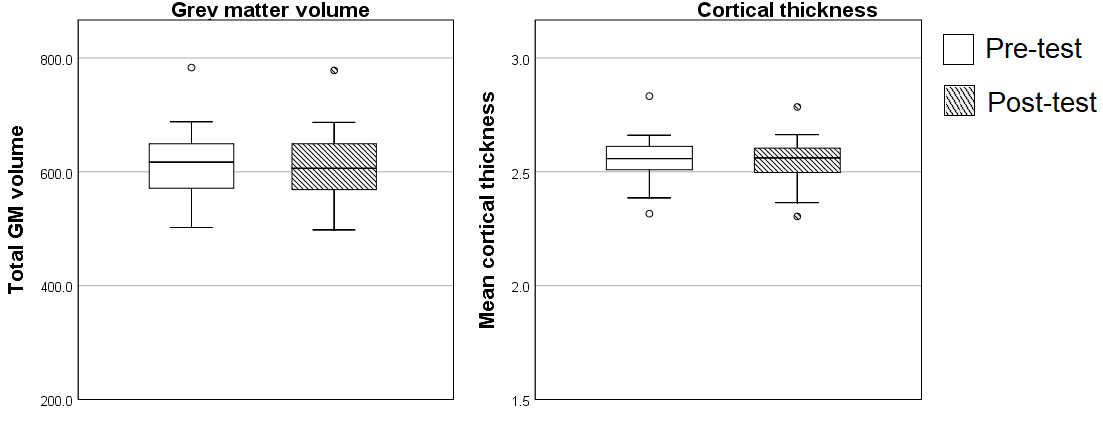


**Figure S3. Effect of session total on grey matter volume.** The figure shows the pre- (white) to post-test (lined) change in total grey matter volume. No statistically significant group or group*session effects were detected.

**Figure S4**


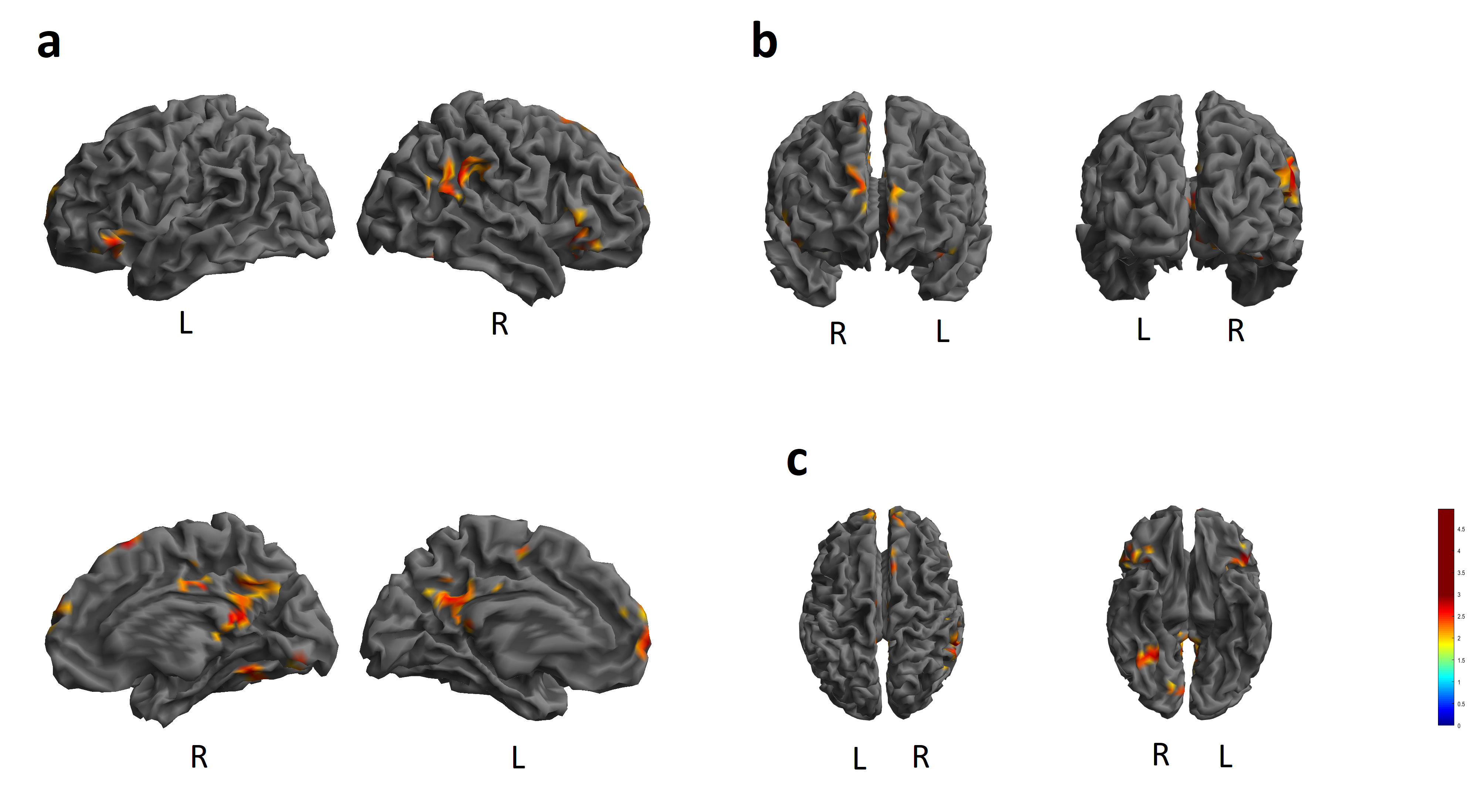


**Figure S4. Effect of load on task-related brain activity.** The figure shows brain areas with higher activity during the 2-back (vs 1-back) compared with the 3-back (vs 1-back) task, across groups. Sagittal (a), coronal (b) and axial (c) view of the brain are reported. The statistically significant clusters are superimposed on a surface rendering on the brain, as provided by SPM 12.

**Figure S5**


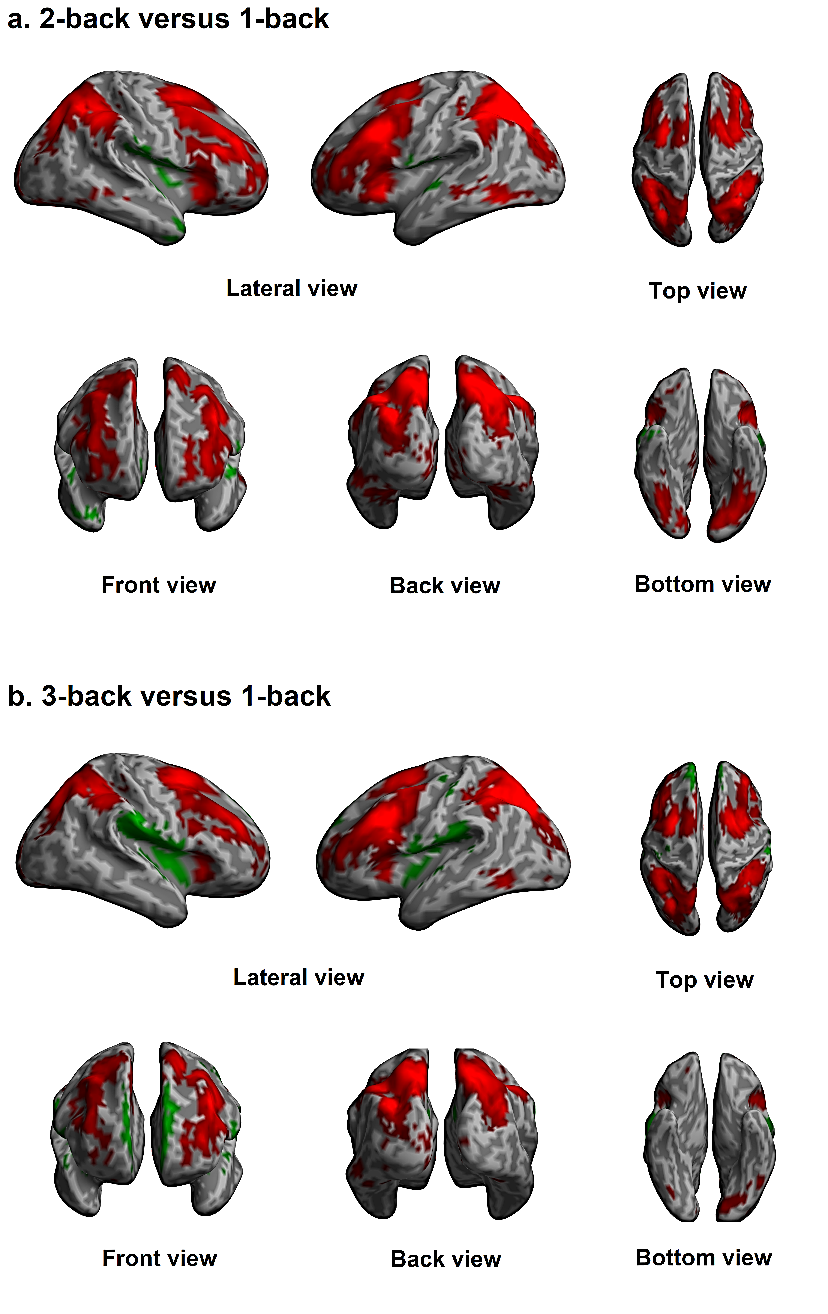


**Figure S5. Effect of the working memory task on brain activity**. The figure represents brain activity during the working-memory task, for the 2-back versus 1-back (a) and 3-back versus 1-back (b) conditions (p-FWE < .05). Red colour indicates areas with higher activity at higher loads (a, 2-back > 1-back; b, or 3-back > 1-back); green indicates areas with higher activity at lower loads (a, 1-back > 2-back; b, 1-back > 3-back). A progressive activation of frontal areas was observable during the 2-back and 3-back relative to the 1-back, and during the 2-back relative to the 3-back. The statistically significant clusters are superimposed on a surface rendering on the brain, as provided by SPM 12.

**REFERENCES**

1 Folstein, M. F., Folstein, S. E. & McHugh, P. R. "Mini-mental state". A practical method for grading the cognitive state of patients for the clinician. *J Psychiatr Res* **12**, 189-198, doi:10.1016/0022-3956(75)90026-6 (1975).

2 Ashburner, J. A fast diffeomorphic image registration algorithm. *Neuroimage* **38**, 95-113, doi:10.1016/j.neuroimage.2007.07.007 (2007).

3 Mazziotta, J. *et al.* A probabilistic atlas and reference system for the human brain: International Consortium for Brain Mapping (ICBM). *Philos Trans R Soc Lond B Biol Sci* **356**, 1293-1322, doi:10.1098/rstb.2001.0915 (2001).

4 Stark, C. E. & Squire, L. R. When zero is not zero: the problem of ambiguous baseline conditions in fMRI. *Proc Natl Acad Sci U S A* **98**, 12760-12766, doi:10.1073/pnas.221462998 (2001).

5 Zhang, Y., Brady, M. & Smith, S. Segmentation of brain MR images through a hidden Markov random field model and the expectation-maximization algorithm. *IEEE Trans Med Imaging* **20**, 45-57, doi:10.1109/42.906424 (2001).

6 Jenkinson, M., Bannister, P., Brady, M. & Smith, S. Improved optimization for the robust and accurate linear registration and motion correction of brain images. *Neuroimage* **17**, 825-841, doi:10.1016/s1053-8119(02)91132-8 (2002).

7 Smith, S. M. Fast robust automated brain extraction. *Hum Brain Mapp* **17**, 143-155, doi:10.1002/hbm.10062 (2002).

8 Schmithorst, V. J. *et al.* Evidence that neurovascular coupling underlying the BOLD effect increases with age during childhood. *Hum Brain Mapp* **36**, 1-15, doi:10.1002/hbm.22608 (2015).

9 Sporns, O. Graph theory methods: applications in brain networks. *Dialogues Clin Neurosci* **20**, 111-121 (2018).

10 Gallen, C. L. & D'Esposito, M. Brain Modularity: A Biomarker of Intervention-related Plasticity. *Trends Cogn Sci* **23**, 293-304, doi:10.1016/j.tics.2019.01.014 (2019).

11 Douw, L., Nieboer, D., van Dijk, B. W., Stam, C. J. & Twisk, J. W. A healthy brain in a healthy body: brain network correlates of physical and mental fitness. *PLoS One* **9**, e88202, doi:10.1371/journal.pone.0088202 (2014).

12 Lebedev, A. V., Nilsson, J. & Lovden, M. Working Memory and Reasoning Benefit from Different Modes of Large-scale Brain Dynamics in Healthy Older Adults. *J Cogn Neurosci* **30**, 1033-1046, doi:10.1162/jocn_a_01260 (2018).

13 Baniqued, P. L. *et al.* Brain Network Modularity Predicts Exercise-Related Executive Function Gains in Older Adults. *Front Aging Neurosci* **9**, 426, doi:10.3389/fnagi.2017.00426 (2017).

14 Adams, E. J., Nguyen, A. T. & Cowan, N. Theories of Working Memory: Differences in Definition, Degree of Modularity, Role of Attention, and Purpose. *Lang Speech Hear Serv Sch* **49**, 340-355, doi:10.1044/2018_LSHSS-17-0114 (2018).

15 Behzadi, Y., Restom, K., Liau, J. & Liu, T. T. A component based noise correction method (CompCor) for BOLD and perfusion based fMRI. *Neuroimage* **37**, 90-101, doi:10.1016/j.neuroimage.2007.04.042 (2007).

16 Jenkinson, M., Beckmann, C. F., Behrens, T. E., Woolrich, M. W. & Smith, S. M. Fsl. *Neuroimage* **62**, 782-790, doi:10.1016/j.neuroimage.2011.09.015 (2012).

17 Power, J. D. *et al.* Functional network organization of the human brain. *Neuron* **72**, 665-678, doi:10.1016/j.neuron.2011.09.006 (2011).

18 Zhu, Y. *et al.* Comparison of Functional Connectivity Estimated from Concatenated Task-State Data from Block-Design Paradigm with That of Continuous Task. *Comput Math Methods Med* **2017**, 4198430, doi:10.1155/2017/4198430 (2017).

19 Rubinov, M. & Sporns, O. Complex network measures of brain connectivity: uses and interpretations. *Neuroimage* **52**, 1059-1069, doi:10.1016/j.neuroimage.2009.10.003 (2010).

20 Rubinov, M. & Sporns, O. Weight-conserving characterization of complex functional brain networks. *Neuroimage* **56**, 2068-2079, doi:10.1016/j.neuroimage.2011.03.069 (2011).

21 Liu, T. T., Nalci, A. & Falahpour, M. The global signal in fMRI: Nuisance or Information? *Neuroimage* **150**, 213-229, doi:10.1016/j.neuroimage.2017.02.036 (2017).

22 Stanley, M. L., Dagenbach, D., Lyday, R. G., Burdette, J. H. & Laurienti, P. J. Changes in global and regional modularity associated with increasing working memory load. *Front Hum Neurosci* **8**, 954, doi:10.3389/fnhum.2014.00954 (2014).

23 Vatansever, D., Menon, D. K., Manktelow, A. E., Sahakian, B. J. & Stamatakis, E. A. Default Mode Dynamics for Global Functional Integration. *J Neurosci* **35**, 15254-15262, doi:10.1523/JNEUROSCI.2135-15.2015 (2015).

24 Yue, Q. *et al.* Brain Modularity Mediates the Relation between Task Complexity and Performance. *J Cogn Neurosci* **29**, 1532-1546, doi:10.1162/jocn_a_01142 (2017).

25 Olivo, G. *et al.* Immediate effects of a single session of physical exercise on cognition and cerebral blood flow: A randomised controlled study of older adults. *NeuroImage*, in press (2020).

26 Blanca, M. J., Alarcon, R., Arnau, J., Bono, R. & Bendayan, R. Non-normal data: Is ANOVA still a valid option? *Psicothema* **29**, 552-557, doi:10.7334/psicothema2016.383 (2017).

27 Ito, P. K. in *Analysis of Variance* Vol. 1 *Handbook of Statistics* (ed P.R. Krishnaiah) Ch. 7, 199-236 (Elsevier B.V., 2020).
